# Correlations in Hard- and Soft-Core Generic Polymer Models

**DOI:** 10.3390/polym15051180

**Published:** 2023-02-26

**Authors:** Qiang Wang

**Affiliations:** Department of Chemical and Biological Engineering, Colorado State University, 1370 Campus Delivery, Fort Collins, CO 80523-1370, USA; q.wang@colostate.edu

**Keywords:** generic polymer models, soft potentials, polymer reference interaction site model theory, invariant degree of polymerization

## Abstract

Generic polymer models capturing the chain connectivity and the non-bonded excluded-volume interactions between polymer segments can be classified into hard- and soft-core models depending on their non-bonded pair potential. Here we compared the correlation effects on the structural and thermodynamic properties of the hard- and soft-core models given by the polymer reference interaction site model (PRISM) theory, and found different behaviors of the soft-core models at large invariant degree of polymerization (IDP) depending on how IDP is varied. We also proposed an efficient numerical approach, which enables us to accurately solve the PRISM theory for chain lengths as large as 10^6^.

While they do not correspond to any chemically specific polymer, generic polymer models are widely used in theoretical and simulation studies in the field of polymer physics as they capture two essential features of all polymers: chain connectivity and non-bonded excluded-volume interactions. Compared to atomistic models that can represent specific polymers used in experiments, molecular simulations of generic models can reach much larger length scales and much longer time scales, and theoretical studies of generic models can also be performed. Depending on whether or not the excluded-volume interactions in generic models prevent complete overlapping of polymer segments, they can be classified into hard-core models (such as those based on the hard-sphere chain model, the Kremer–Grest model [1], and the various self- and mutual-avoiding walk models on a lattice) and soft-core models (such as those used in the dissipative particle dynamics (DPD) simulation [2], fast Monte Carlo simulations [3,4,5,6,7], field-theoretic simulation (FTS) [8], variants of FTS under the partial saddle-point approximation [9], single-chain-in-mean-field simulation [10] and hybrid particle field molecular dynamics simulation [11] both under the quasi-instantaneous field approximation [10]). Taking the study of polymer melts as an example, while hard-core models have been used in conventional molecular simulations for a long time, they have the disadvantage that their chain lengths *N* used in such simulations (as limited by the computational cost) are too short compared to those in typical experiments; in other words, such conventional simulations significantly exaggerate the fluctuations in polymer melts compared to experiments [6,7,12]. In contrast, simulations of the more recently proposed soft-core models can readily reach the extent of fluctuations in typical experiments by increasing the chain number density (or equivalently the segment number density *ρ* at finite *N*) instead of *N* [6,7,12].

In this Letter we focus on a simple but important class of generic models for compressible homopolymer melts (or equivalently homopolymer solutions in an implicit solvent) in the continuum, with the excluded-volume interaction between polymer segments described by a short-range, isotropic and purely repulsive pair potential *βu*^nb^(*r*), where *β* ≡ 1/*k_B_T* with *k_B_* being the Boltzmann constant and *T* the thermodynamic temperature of the system; this is the basis of more complicated polymer models having attractions and/or more species. The hard- and soft-core models can then be classified according to whether $\int dr\beta u^{\mathrm{nb}}(|r|)$ diverges or not. This classification becomes clear after we write the total dimensionless non-bonded interaction energy for a system of *n* chains each having *N* segments in volume *V* under the commonly used pairwise additivity as

$$\beta U^{\mathrm{nb}}=\sum_{i}\sum_{j>i}\beta u^{\mathrm{nb}}(|r_{i}-r_{j}|)=\left(\rho^{2}/2\right)\int drdr^{\prime }\phi (r)\beta u^{\mathrm{nb}}(\left|r-r^{\prime }\right|)\phi (r^{\prime })-\left(nN/2\right)\beta u^{\mathrm{nb}}(0),$$

where **r***_i_* denotes the spatial position of the *i*th segment in the system, $\phi (r)\equiv \sum_{i=1}^{nN}\delta (r-r_{i})/\rho$ is the segment volume fraction at **r**, and the last term deducting the self-interaction of segments gives an unimportant constant; while molecular simulations of this system can be performed at finite *ρ* ≡ *nN*/*V* for any *βu*^nb^(*r*) (along with a chain-connectivity model), for a ***homogeneous*** system (i.e., ϕ(r)=1) the widely used polymer self-consistent field (SCF) theory [13] gives the dimensionless internal energy per chain due to the non-bonded interaction $\beta U^{\mathrm{nb}}/n=\left(N\rho /2\right)\int dr\beta u^{\mathrm{nb}}(\left|r\right|)-\left(N/2\right)\beta u^{\mathrm{nb}}(0)$ (due to its mean-field approximation that neglects the system fluctuations and correlations), which diverges if $\int dr\beta u^{\mathrm{nb}}(\left|r\right|)$ does (i.e., for the hard-core models). It is then clear that the SCF theory can only be applied to soft-core models, where one can define the dimensionless excluded-volume interaction parameter *ε* > 0 via *u*^nb^(*r*) = *εu*_0_(*r*) with the normalized pair potential *u*_0_(*r*) satisfying $\int dr\beta u_{0}(\left|r\right|)=1$. Another necessary condition for applying the SCF theory (i.e., having finite *βU*^nb^/*n*) is *ε* ∝ *ρ^−^*^1^.

Here we compare the correlation effects on the structural and thermodynamic properties of hard- and soft-core generic polymer models, which has rarely been reported [14], to further reveal their differences. For this purpose we choose the polymer reference interaction site model (PRISM) theory proposed by Schweizer and Curro [15], which has been applied to many polymeric systems, including homopolymer melts, solutions, blends, block copolymers, nanocomposites, polyelectrolytes, etc. [16,17,18,19] It can be considered as the most successful molecular-level theory to date for studying the correlations in homogeneous polymeric systems.

For the above homopolymer melts, the PRISM equation is given by

(1) $$\hat{h}=N\hat{\omega }\hat{c}\left(N\hat{\omega }+\bar{\rho }\hat{h}\right),$$

where $h(\bar{r})$ is the interchain total segment pair correlation function (PCF) with $\bar{r}\equiv r/\sigma$ and *σ* the segment diameter (i.e., the range of *βu*^nb^(*r*)), $\omega (\bar{r})$ is the normalized (i.e., $4\pi \int_{0}^{\infty }d{\bar{\mathrm{rr}}}^{2}\omega (\bar{r})=1$) intrachain segment PCF, $c(\bar{r})$ is the interchain direct segment PCF, $\hat{f}\equiv \left(4\pi /q\right)\int_{0}^{\infty }d\bar{\mathrm{rr}}f(\bar{r})\sin\left(q\bar{r}\right)$ denotes the 3D Fourier transform of a radial function $f(\bar{r})$ with *q* being the wavenumber (in units of 1/*σ*), and $\bar{\rho }\equiv nN\sigma^{3}/V$ is the dimensionless segment number density. For given *N*, $\bar{\rho }$ and *ω*, to solve for both *h* and *c*, a closure providing an approximate relation between them is needed; here we take the commonly used Percus–Yevick (PY) closure [20]

(2) $$c(\bar{r})=[1-\exp(\beta u^{\mathrm{nb}}(\bar{r}))]\left(1+h(\bar{r})\right),$$

which works well for our class of generic models where $\beta u^{\mathrm{nb}}(\bar{r})$ is short-range and purely repulsive.

To be more specific, we consider two commonly used generic polymer models: the tangent hard-sphere chain (THSC) and the DPD models; the former is a hard-core model that uses $\exp(-\beta u^{b}(\bar{r}))=\delta (\bar{r}-1)/4\pi$ with $\beta u^{b}(\bar{r})$ specifying the dimensionless bonded potential between two adjacent segments on the same chain and the hard-sphere (HS) potential $\beta u^{\mathrm{HS}}(\bar{r})\to \infty$ for $\bar{r}<1$ and 0 otherwise as $\beta u^{\mathrm{nb}}(\bar{r})$, and the latter is a soft-core model that uses $\beta u^{b}(\bar{r})=2{\bar{r}}^{2}$ and the DPD potential $\beta u^{\mathrm{DPD}}(\bar{r})=\left(a/2\right){\left(1-\bar{r}\right)}^{2}$ for $\bar{r}<1$ and 0 otherwise as $\beta u^{\mathrm{nb}}(\bar{r})$ with the dimensionless interaction parameter $a=15\varepsilon /\pi =75/\bar{\rho }$ chosen to mimic the compressibility of water [2]. In the thermodynamic limit, the structural and thermodynamic properties of these two models are controlled only by *N* and $\bar{\rho }$; typically, molecular simulations of the DPD model uses $\bar{\rho }=3$ or 5.

Finally, we note that $\hat{\omega }$ is needed as an input for PRISM calculations. While in general the chain conformations characterized by $\hat{\omega }$ depend on $\bar{\rho }$, for simplicity here we use the ideal-chain conformations by setting $\hat{\omega }$ to ${\hat{\omega }}_{\mathrm{id}}=\left[N-\tilde{B}(q)\left(2+N\tilde{B}(q)-2{\tilde{B}}^{N}(q)\right)\right]/N^{2}{\left(1-\tilde{B}(q)\right)}^{2}$ with $\tilde{B}(q)\equiv \int d\bar{r}\exp(iq\cdot \bar{r})\exp\left(-\beta u^{b}(\left|\bar{r}\right|)\right)/\int d\bar{r}\exp\left(-\beta u^{b}(\left|\bar{r}\right|)\right)=\sin q/q$ for the THSC model and $\exp\left(-q^{2}/8\right)$ for the DPD model, where $\bar{r}\equiv r/\sigma$, **q** is the wave vector for the 3D Fourier transform, and *q*=|**q**|.

For the two generic models that we consider here, the PY closure gives $c(\bar{r}\geq 1)=0$. Since all previously reported numerical methods for PRISM calculations [21,22,23,24] are not optimal in this case, we first propose an efficient numerical approach as follows. We uniformly discretize the real-space interval [0, 1] into *m* subintervals (thus $\left[0,{\bar{r}}_{c}\right]$ into $M\equiv m{\bar{r}}_{c}$ subintervals) each of length $\Delta \bar{r}\equiv 1/m$, where ${\bar{r}}_{c}\gg 1$ denotes the real-space cut-off, and take $c({\bar{r}}_{i}\equiv i\Delta \bar{r})$ (*i* = 0, …, *m*−1 for the DPD model and *i* = 0, …, *m* with ${\bar{r}}_{m}=1^{-}$ for the THSC model) as the independent variables to be solved. Our approach has three steps:

- Given the initial guess of the independent variables and $c(\bar{r}\geq 1)=0$, for the DPD model we calculate $\hat{c}(q_{j}\equiv j\Delta q)=(4\pi /q_{j})\int_{0}^{{\bar{r}}_{c}}d\bar{\mathrm{rr}}c(\bar{r})\sin\left(q_{j}\bar{r}\right)$ (*j* = 1, …, *M*−1) via the discrete sine transform of type I (DST) [25], which has the computational complexity of *O*(*M*ln*M*) and gives Δq=mπ/M, the reciprocal-space cut-off *q_c_* = *q_M_* = *mπ* and $\hat{c}(q_{c})=0$; for the THSC model, due to the discontinuities in both $c(\bar{r})$ and its 1st-order derivative at $\bar{r}=1$, we use an auxiliary function $\tilde{c}(\bar{r})\equiv c(\bar{r})-c_{1}-c_{1}^{\prime }\left(\bar{r}-1\right)$ for $\bar{r}\leq 1^{-}$ and $c(\bar{r})$ otherwise with $c_{1}\equiv c(\bar{r}=1^{-})$ and $c_{1}^{\prime }\equiv \left(dc/d\bar{r}\right)(\bar{r}=1^{-})$ (calculated via the fourth-order backward finite-difference formula [26]), which is continuous in both its value and 1st-order derivative, to calculate $\hat{c}(q_{j})=(4\pi /q_{j})\left(\int_{0}^{{\bar{r}}_{c}}d\bar{\mathrm{rr}}\tilde{c}(\bar{r})\sin(q_{j}\bar{r})+\left\{c_{1}(\sin q_{j}-q_{j}\cos q_{j})+c_{1}^{\prime }[2\left(\cos q_{j}-1\right)/q_{j}+\sin q_{j}]\right\}/q_{j}{}^{2}\right)$ (*j* = 1, …, *M*) via the DST, which gives $\hat{c}(q_{c})=\left[8({\left(-1\right)}^{m}-1)c_{1}^{\prime }-4{\left(-1\right)}^{m}\pi^{2}m^{2}c_{1}\right]/\pi^{3}m^{4}$. We also calculate ${\hat{c}}_{0}\equiv \hat{c}(q_{0})=4\pi \int_{0}^{1}d{\bar{\mathrm{rr}}}^{2}c(\bar{r})$ for the DPD model and ${\hat{c}}_{0}=4\pi \int_{0}^{1^{-}}d{\bar{\mathrm{rr}}}^{2}c(\bar{r})$ for the THSC model via the Romberg integration (RI) [27].
- We calculate $\hat{\gamma }(q_{j})=\left[N^{2}{\hat{\omega }}^{2}(q_{j})/\left(1-N\bar{\rho }\hat{\omega }(q_{j})\hat{c}(q_{j})\right)-1\right]\hat{c}(q_{j})$ (*j* = 0, …, *M*) obtained from Equation (1) with $\gamma (\bar{r})\equiv h(\bar{r})-c(\bar{r})$ being the interchain indirect segment PCF (note that ${\hat{\gamma }}_{c}\equiv \hat{\gamma }(q_{c})=0$ for the DPD model while ${\hat{\gamma }}_{c}\neq 0$ for the THSC model), then for the DPD model $\gamma ({\bar{r}}_{j})=\left(1/2\pi^{2}{\bar{r}}_{j}\right)\int_{0}^{q_{c}}dqq\hat{\gamma }\sin\left(q{\bar{r}}_{j}\right)$ (*j* = 1, …, *M*−1) via the DST (which gives $\gamma ({\bar{r}}_{c})=0$); for the THSC model, we use another auxiliary function $\tilde{\gamma }(q)\equiv \hat{\gamma }-{\hat{\gamma }}_{c}$ to calculate $\gamma ({\bar{r}}_{j})=\left(1/2\pi^{2}{\bar{r}}_{j}\right)\left[\int_{0}^{q_{c}}dqq\tilde{\gamma }(q)\sin\left(q{\bar{r}}_{j}\right)-{\left(-1\right)}^{j}\pi m^{2}{\hat{\gamma }}_{c}/j\right]$ (*j* = 1, …, *M*) via the DST, which gives $\gamma ({\bar{r}}_{c})=-{\left(-1\right)}^{j}m^{3}{\hat{\gamma }}_{c}/2\pi M^{2}$. We also calculate $\gamma ({\bar{r}}_{0})=\left(1/2\pi^{2}\right)\int_{0}^{q_{c}}dqq^{2}\hat{\gamma }$ for both models via the RI.
- We calculate $h({\bar{r}}_{i})=\gamma ({\bar{r}}_{i})+c({\bar{r}}_{i})$ (*i* = 0, …, *m*–1 for the DPD model and *i* = 0, …, *m* for the THSC model), then use the residual errors of Equation (2) (which becomes $h({\bar{r}}_{i})=-1$ for the THSC model) to converge the independent variables via the Anderson mixing [28], which has the computational complexity of *O*(*m*) and can quickly converge a large set of nonlinear equations to a high accuracy.

We use the convergence criterion of *ε_c_* < 10^−10^ with *ε_c_* denoting the maximum absolute value of the residual errors of the PY closure over all ${\bar{r}}_{i}$ (*i* = 0, …, *m*–1 for the DPD model and *i* = 0, …, *m* for the THSC model), and choose the values of *m* (=4096 for the THSC model and 512 for the DPD model) and ${\bar{r}}_{c}$ ($\approx 10\sqrt{N}$ if *N* < 100 and $2\sqrt{N}$ otherwise, rounded to the nearest integer, to capture the correlation-hole effect [29]) such that the discretization errors are comparable to *ε_c_*. Our numerical approach has the least number of independent variables to be iteratively solved, greatly reduces *m* (thus *M*) both by analytically treating the discontinuities in the THSC model and by taking the inverse Fourier transform only for $\hat{\gamma }$ (which decays toward 0 with increasing *q* much faster than both $\hat{c}$ and $\hat{h}$), and is essential for us to accurately solve the PRISM-PY theory for *N* as large as 10^6^ (where for the THSC model *M* is about 8.2 × 10^6^!). To the best of our knowledge, analytically treating the discontinuities caused by the HS potential has not been reported in numerical calculations of even the widely studied Ornstein–Zernike (OZ) equation [30] (to which Equation (1) reduces for *N* = 1); in Supplemental Material we show that our numerical approach gives several orders of magnitude more accurate results than pyPRISM [19], a recently developed Python-based open-source framework for PRISM calculations.

In the limit of *N*→∞ and *σ*→0 at finite root-mean-square end-to-end distance of the ideal chain $R_{e,0}\equiv \sqrt{N-1}\sigma$, the THSC model becomes the hard-core Gaussian thread model [31] (HC CGC-*δ*, where *R_e_*_,0_ is taken as the unit of length); to compare the PRISM-PY results of these two models, we define two dimensionless parameters: $C_{0}\equiv N^{2}{\hat{c}}_{0}\sigma^{3}/R_{e,0}{}^{3}$ and the invariant degree of polymerization [32] $\bar{N}\equiv {\left(nR_{e,0}{}^{3}/V\right)}^{2}$ ; $\bar{N}$ controls the fluctuations in polymer melts, and for the THSC model it is easy to show that $\bar{N}$ ∝ *N* at large *N*. Figure 1a shows how *C*_0_ varies with $\bar{N}$ for the THSC and HC CGC-*δ* models; for the latter model, $\bar{N}$ is the only parameter, the PRISM-PY equation is given by Equation (18) in our previous work [14] and the corresponding numerical results for $\bar{N}$ ≥ 100 are shown in figure 8b there. We see that, while −*C*_0_ increases monotonically with increasing $\bar{N}$ for the HC CGC-*δ* model, it exhibits a minimum for the THSC model. At given $\bar{N}$ due to its *N*→∞ the HC CGC-*δ* model corresponds to the limit of $\overset{¯}{\rho }=\sqrt{\overset{¯}{N}}N/{\left(N-1\right)}^{3/2}\to 0$ of the THSC model as implied in Figure 1a. At large $\bar{N}$ , we see that $-C_{0}\propto \sqrt{\overset{¯}{N}}$ in all cases. This is in accordance with an asymptotic value of ${\hat{c}}_{0}<0$ at given $\bar{\rho }$ for the THSC model, while ${\hat{c}}_{0}\to 0$ for the HC CGC-*δ* model. Figure 1a also shows that the DPD model at $\bar{\rho }=3$ gives qualitatively the same behavior of *C*_0_ vs. $\bar{N}$ as that for the THSC model.

With the normalized isothermal compressibility ${\overset{¯}{\kappa }}_{T}\equiv \rho_{c}\kappa_{T}/\beta =1/\left(1-\sqrt{\overset{¯}{N}}C_{0}\right)$ given by the compressibility equation, where *ρ_c_* ≡ *n*/*V* is the chain number density and $\kappa_{T}\equiv -{\left(\partial V/\partial P\right)}_{n,\beta }/V$ is the isothermal compressibility with *P* denoting the system pressure, Figure 1b presents essentially the same data as in Figure 1a, but in a way that can be compared with real polymers used in experiments. As shown in figure 2 of our previous work [14], ${\overset{¯}{\kappa }}_{T}\overset{¯}{N}\approx 1.38$ for polyethylene (at 180 °C) and 0.119 for polystyrene (at 280 °C), independent of their $\bar{N}$ ≥10^3^; this is consistent with $-C_{0}\propto \sqrt{\overset{¯}{N}}$ at large $\bar{N}$ shown in Figure 1a. On the other hand, while ${\overset{¯}{\kappa }}_{T}\overset{¯}{N}\propto \overset{¯}{N}$ is expected for very small $\bar{N}$, the smallest $\bar{N}$ (given by *N* = 2) is about 0.0025, 0.090 and 0.95, respectively, for the THSC model at $\bar{\rho }=0.1$ and 0.6 and the DPD model at $\bar{\rho }=3$. Clearly, both hard- and soft-core models can be used to describe the excluded-volume interactions in real polymers, and experimental values of ${\bar{\kappa }}_{T}$ can be achieved by adjusting $\bar{\rho }$, for example, in the THSC and DPD models. We attribute the largest ${\bar{\kappa }}_{T}$ at the same $\bar{N}$ given by the HC CGC-*δ* model to its *σ*→0, and note that the DPD model at $\bar{\rho }=3$ is actually “harder” (i.e., more difficult to compress) than the hard-core models studied here.

Figure 1c shows that at large $\bar{N}$, the dimensionless excess (virial) pressure due to the interchain repulsion $\beta R_{e,0}{}^{3}P_{\mathrm{ex}}=\left(2\pi /3\right)\left[\overset{¯}{N}N^{2}/{\left(R_{e,0}/\sigma \right)}^{3}\right]\left(h\left(\overset{¯}{r}=1\right)+1\right)$ scales with ${\overset{¯}{N}}^{3/2}$ for the THSC model; this is due to the same scaling of *R_e_*_,0_^3^ with *N* and also found for the HC CGC-*δ* model (where $\beta R_{e,0}{}^{3}P_{\mathrm{ex}}=-C_{0}\overset{¯}{N}/2$). We also see that the HC CGC-*δ* model gives a much smaller *βR_e_*_,0_^3^*P*_ex_ than the THSC model at the same $\bar{N}$ , again due to its *σ*→0. Figure 1c further shows that at large $\bar{N}$ , the DPD model at $\bar{\rho }=3$ gives the same scaling of $\beta R_{e,0}{}^{3}P_{\mathrm{ex}}=-\left(2\pi /3\right)\left[\overset{¯}{N}N^{2}/{\left(R_{e,0}/\sigma \right)}^{3}\right]\int_{0}^{1}d{\bar{\mathrm{rr}}}^{3}(h(\overset{¯}{r})+1)(d\beta u^{\mathrm{DPD}}(\overset{¯}{r})/d\overset{¯}{r})$ with $\bar{N}$ as the hard-core models; at the same $\bar{N}$ , it has even the largest $\beta R_{e,0}{}^{3}P_{\mathrm{ex}}$, in accordance with its smallest ${\bar{\kappa }}_{T}$ shown in Figure 1b.

Note that for both the THSC and DPD models, $\bar{N}$ is varied by changing *N* at fixed $\bar{\rho }$ in Figure 1, which makes $\bar{N}$ and *N* to be approximately on the same order; it is therefore very difficult, if possible at all, to reach via this way in molecular simulations even a relatively small $\bar{N}$-value of 10^4^ used in experiments. As aforementioned, molecular simulations of soft-core models can readily reach $\bar{N}$-values used in experiments by increasing $\bar{\rho }$ at fixed *N* [6,7,12]. For the DPD model at large $\bar{\rho }$, $\beta u^{\mathrm{DPD}}(\bar{r})=\left(75/2\bar{\rho }\right){\left(1-\bar{r}\right)}^{2}\approx 0$ and the PY closure approaches the random-phase approximation (RPA) closure [33,34] $c^{\mathrm{RPA}}(\bar{r})=-\beta u^{\mathrm{DPD}}(\bar{r})$, which gives $c_{0}^{\mathrm{RPA}}=-75/2\bar{\rho }$ and ${\hat{c}}_{0}^{\mathrm{RPA}}=-5\pi /\bar{\rho }$ independent of *N*. We then obtain ${\bar{\kappa }}_{T}^{\mathrm{RPA}}=1/\left(1+5\pi N\right)$ from the compressibility equation. Figure 2a shows ${\bar{\kappa }}_{T}$ vs. $1/\bar{\rho }$ obtained via the compressibility equation from our PRISM-PY calculations of the DPD model at various *N*, where each curve exhibits a minimum with its location shifting to smaller $1/\bar{\rho }$ with increasing *N* and the intercept of each (extrapolated) curve with the left axis (i.e., in the limit of $\bar{\rho }\to \infty$) gives the corresponding ${\bar{\kappa }}_{T}^{\mathrm{RPA}}$. Clearly, the difference between ${\bar{\kappa }}_{T}$ and ${\bar{\kappa }}_{T}^{\mathrm{RPA}}$ is entirely due to that between the PY and RPA closures.

A *C*_0_ vs. $\bar{N}$ plot (not shown) can be obtained from Figure 2a for the DPD model. In particular, the RPA closure gives $-C_{0}^{\mathrm{RPA}}=5\pi N/\sqrt{\overset{¯}{N}}$, indicating that $-C_{0}\propto {\overset{¯}{N}}^{-1/2}$ at large $\bar{N}$; this is in clear contrast to $-C_{0}\propto {\overset{¯}{N}}^{1/2}$ for the hard-core models and the DPD model at $\bar{\rho }=3$ shown in Figure 1a, but consistent with the soft-core Gaussian thread (SC CGC-*δ*) model (which is equivalent to the well-known Edwards model [35]) shown in figure 8a of our previous work [14], where *N*→∞ and *σ*→0 at finite *R_e_*_,0_ and $\beta u^{\mathrm{nb}}(r)=\left(\bar{\kappa }/N^{2}\rho_{c}\right)\delta (r)$ is used with a ***finite*** dimensionless parameter $\bar{\kappa }>0$ controlling the repulsion strength between polymer segments. The behavior of soft-core models at large $\bar{N}$ , therefore, depends on how $\bar{N}$ is varied, i.e., whether by changing *N* at fixed $\bar{\rho }$ (thus the excluded-volume interaction parameter *ε* is fixed) or by changing $\bar{\rho }$ at fixed *N* (thus *ε* is also varied as $\propto {\bar{\rho }}^{-1}$); in the former case correlations exist even in the limit of *N*→∞, while in the latter case the SCF theory becomes exact in the limit of $\bar{\rho }\to \infty$ (at finite *N*) where neither fluctuations nor correlations exist.

As aforementioned, with increasing $\bar{\rho }$ at fixed *N*, the PY closure approaches the RPA closure, which gives ${\hat{c}}^{\mathrm{RPA}}=-\left(5\pi /\bar{\rho }\right)\beta {\hat{u}}_{0}$ thus ${\hat{h}}^{\mathrm{RPA}}=-5\pi N^{2}{\left({\hat{\omega }}_{\mathrm{id}}^{\mathrm{DPD}}\right)}^{2}\beta {\hat{u}}_{0}/\bar{\rho }\left(1+5\pi N{\hat{\omega }}_{\mathrm{id}}^{\mathrm{DPD}}\beta {\hat{u}}_{0}\right)$ according to Equation (1). In the limit of $\bar{\rho }\to \infty$, we have $c^{\mathrm{RPA}}(\bar{r})\to 0$ and $h^{\mathrm{RPA}}(\bar{r})\to 0$, thus the SCF results of $\beta \sigma^{3}P_{\mathrm{ex}}^{\mathrm{SCF}}/\bar{\rho }=5\pi /2$ and $\beta u_{c,\mathrm{ex}}^{\mathrm{SCF}}/N=5\pi /2$ independent of *N*, where $\beta u_{c,\mathrm{ex}}=75\pi N\int_{0}^{1}d{\bar{\mathrm{rr}}}^{2}\left(h(\bar{r})+1\right){\left(1-\bar{r}\right)}^{2}$ denotes the dimensionless excess internal energy per chain due to the interchain repulsion. On the other hand, the differences between the SCF and RPA results as given by $\beta \sigma^{3}\left(P_{\mathrm{ex}}^{\mathrm{SCF}}-P_{\mathrm{ex}}^{\mathrm{RPA}}\right)/a\bar{\rho }=-\left(2\pi /3\right)\bar{\rho }\int_{0}^{1}d{\bar{\mathrm{rr}}}^{3}h^{\mathrm{RPA}}(\bar{r})\left(1-\bar{r}\right)$ and $\beta \left(u_{c,\mathrm{ex}}^{\mathrm{SCF}}-u_{c,\mathrm{ex}}^{\mathrm{RPA}}\right)/aN=-\pi \bar{\rho }\int_{0}^{1}d\bar{r}{\bar{r}}^{2}h^{\mathrm{RPA}}(\bar{r}){\left(1-\bar{r}\right)}^{2}$ are independent of $\bar{\rho }$.

Finally, Figure 2b shows that $\beta \sigma^{3}\left(P_{\mathrm{ex}}-P_{\mathrm{ex}}^{\mathrm{RPA}}\right)/a\bar{\rho }\propto {\bar{\rho }}^{-1}$ at large $\bar{\rho }$; note that $P_{\mathrm{ex}}>P_{\mathrm{ex}}^{\mathrm{RPA}}$ at large $\bar{\rho }$ while $P_{\mathrm{ex}}<P_{\mathrm{ex}}^{\mathrm{RPA}}$ at small $\bar{\rho }$, which leads to the cusp of each curve shown in the figure with its location (i.e., the $\bar{\rho }$-value at which $P_{\mathrm{ex}}=P_{\mathrm{ex}}^{\mathrm{RPA}}$) increasing with increasing *N* (the cusp at *N* = 1 is located around $\bar{\rho }=2.6$). We also note that $\beta \sigma^{3}\left(P_{\mathrm{ex}}^{\mathrm{SCF}}-P_{\mathrm{ex}}^{\mathrm{RPA}}\right)/a\bar{\rho }\approx 0.0327$, 0.119 and 0.182 for *N* = 1, 10 and 100. Therefore, with increasing $\bar{\rho }$, both $\beta \sigma^{3}P_{\mathrm{ex}}/\bar{\rho }$ and $\beta \sigma^{3}P_{\mathrm{ex}}^{\mathrm{RPA}}/\bar{\rho }$ approach $\beta \sigma^{3}P_{\mathrm{ex}}^{\mathrm{SCF}}/\bar{\rho }$. Similar results are found for $\beta \left(u_{c,\mathrm{ex}}-u_{c,\mathrm{ex}}^{\mathrm{RPA}}\right)/aN$ as shown in Figure 2c, where $u_{c,\mathrm{ex}}<u_{c,\mathrm{ex}}^{\mathrm{RPA}}$ at large $\bar{\rho }$ while $u_{c,\mathrm{ex}}>u_{c,\mathrm{ex}}^{\mathrm{RPA}}$ at small $\bar{\rho }$ (with the cusp at *N* = 1 located around $\bar{\rho }=2.2$); also note that $\beta \left(u_{c,\mathrm{ex}}^{\mathrm{SCF}}-u_{c,\mathrm{ex}}^{\mathrm{RPA}}\right)/aN\approx 0.144$, 0.253 and 0.322 for *N*=1, 10 and 100. In particular, the PRISM-RPA theory with ${\hat{\omega }}_{\mathrm{id}}^{\mathrm{DPD}}$ is equivalent to the Gaussian-fluctuation theory neglecting non-Gaussian fluctuations in the system and gives a correction $\propto {\bar{\rho }}^{-1}$ to the SCF result, while the PRISM-PY theory captures non-Gaussian fluctuations in an approximate way and gives a leading-order correction $\propto {\bar{\rho }}^{-2}$ to the Gaussian-fluctuation result. These are consistent with our previous study of compressible [36] and incompressible [37] homopolymer melts using fast lattice Monte Carlo simulations [6,7]. Given this and the agreement of our Figure 1b with experimental results at large $\bar{N}$ , we do not expect that the use of more accurate $\hat{\omega }$ can qualitatively change our PRISM-PY results here.

To summarize, we have compared the correlation effects on the structural and thermodynamic properties of hard-core models (i.e., the THSC model and its limit of *N*→∞ at finite *R_e_*_,0_ (or equivalently $\bar{\rho }\to 0$ at given $\bar{N}$ ), the HC CGC-*δ* model [31]) and soft-core models (i.e., the DPD model and its limit of *N*→∞ at finite *R_e_*_,0_, the Edwards model [35]) for compressible homopolymer melts (or equivalently homopolymer solutions in an implicit solvent) given by the PRISM-PY theory. The behavior of soft-core models at large $\bar{N}$ depends on how $\bar{N}$ is varied, i.e., whether by changing *N* at fixed $\bar{\rho }$ (thus *ε* is fixed) or by changing $\bar{\rho }$ at fixed *N* (thus *ε* is also varied as being inversely proportional to $\bar{\rho }$). In the former case, correlations exist even in the limit of *N*→∞, and both the hard-core and the DPD models give $-C_{0}\propto {\overset{¯}{N}}^{1/2}$ at large $\bar{N}$ , consistent with real polymers used in experiments; it is, however, very difficult to reach via this way in molecular simulations even a relatively small $\bar{N}$ -value of 10^4^ used in experiments. This problem is solved in the latter case, where the widely used polymer SCF theory becomes exact in the limit of $\bar{\rho }\to \infty$ (at finite *N*), the Gaussian-fluctuation theory gives a correction $\propto {\bar{\rho }}^{-1}$ to the SCF result, and the PRISM-PY theory captures non-Gaussian fluctuations in the system in an approximate way and gives a leading-order correction $\propto {\bar{\rho }}^{-2}$ to the Gaussian-fluctuation result, consistent with our previous simulations [36,37]. The soft-core models, however, give $-C_{0}\propto {\overset{¯}{N}}^{-1/2}$ at large $\bar{N}$ , suggesting that it would be difficult, if possible at all, for the various recently proposed simulation methods [3,4,5,6,7,8,9,10,11] to capture both the fluctuations and correlations in experimental systems. We also proposed an efficient numerical approach, which enables us to accurately solve the PRISM-PY theory for *N* as large as 10^6^; numerical calculations of such theories can, therefore, capture both the fluctuations and correlations in experimental systems.

## Figures and Tables

**Figure 1:**
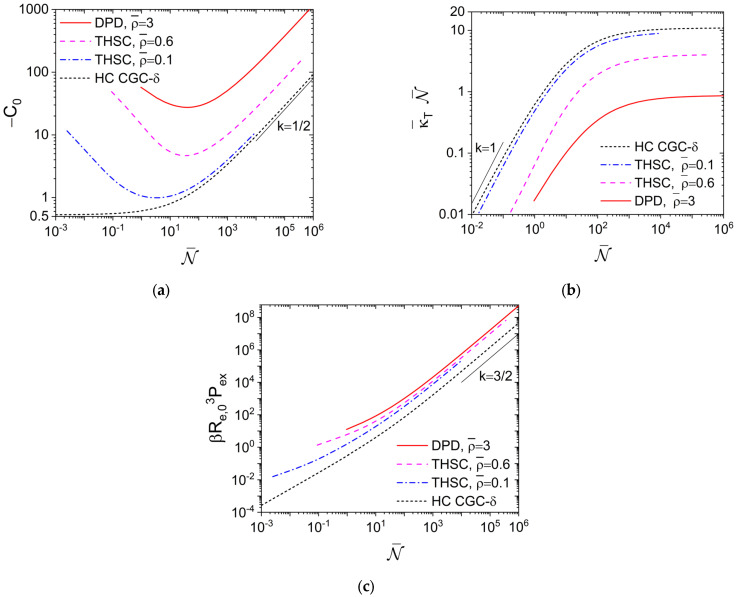
Logarithmic plot of (**a**) $C_{0}$, (**b**) normalized isothermal compressibility ${\bar{\kappa }}_{T}$, and (**c**) excess (virial) pressure due to the interchain repulsion *P*_ex_ vs. the invariant degree of polymerization $\bar{N}$ of various models. The *k*-value gives the slope of the corresponding straight line. See the main text for more details.

**Figure 2:**
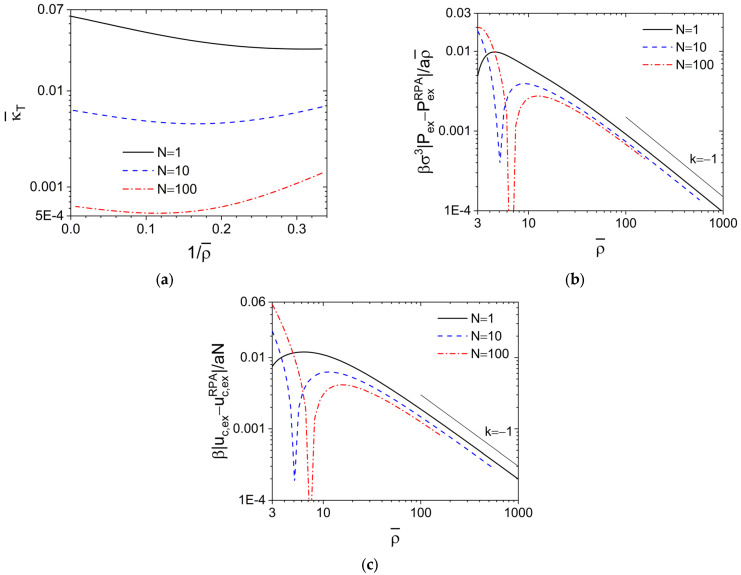
(**a**) Semi-logarithmic plot of the normalized isothermal compressibility ${\bar{\kappa }}_{T}$ and logarithmic plot of the difference between (**b**) the excess (virial) pressure *P*_ex_ and (**c**) the excess internal energy per chain *u_c_*_,ex_ due to the interchain interactions given by the PRISM-PY calculations and that by the PRISM-RPA calculations of the DPD model at various chain lengths *N*. The *k*-value gives the slope of the corresponding straight line. See the main text for more details.

## Data Availability

The in-house code written in C and the data presented in this study are available upon reasonable request from the corresponding author.

## Supplementary Materials

The following supporting information can be downloaded at: https://www.mdpi.com/article/10.3390/polym15051180/s1, Figure S1: Logarithmic plot of the numerical errors given by pyPRISM and our approach for the HS model; Table S1: List of variables used in the main text.
