# Supplementary material for: Correlations in Hard- and Soft-Core Generic Polymer Models
Source: Polymers (Basel). 2023 Feb 26;15(5):1180. doi: 10.3390/polym15051180 (PMC10007592; doi:10.3390/polym15051180)
Supplement: Supplementary file 1 [file polymers-15-01180-s001.zip › polymers-2235843-supplementary.pdf]

Supplemental Material: Correlations in Hard- and Soft-Core Generic Polymer Models

Qiang Wang[*]

Department of Chemical and Biological Engineering, Colorado State University

1370 Campus Delivery, Fort Collins, CO 80523-1370

Here we follow the same notation as in our main text (see Table S1 below) and quantitatively compare the performance of our numerical approach proposed in the main text with pyPRISM[1]. pyPRISM is a recently developed Python-based open-source framework for PRISM calculations. It uniformly discretizes the real space as in our proposed numerical approach, but takes the values of $\gamma(\bar{r})$ at the discretized positions as the independent variables to be solved. It also has three steps: (I) Given the initial guess of the independent variables, it calculates $c(\bar{r})$ from the closure and then $\hat{c}$ via the direct 3D Fourier transform of $c(\bar{r})$. (II) It calculates $\hat{h} = N^2\hat{\omega}^2\hat{c}/(1 - N\bar{\rho}\hat{\omega}\hat{c})$ from Eq. (1), then $h(\bar{r})$ via the direct inverse Fourier transform of $\hat{h}$. (III) It calculates $\gamma(\bar{r}) = h(\bar{r}) - c(\bar{r})$, then uses a Newton-Krylov based iteration by default to converge the independent variables. Clearly, in our case of $c(\bar{r} \geq 1) = 0$, the number of its independent variables is $\bar{r}_c$ times larger than ours, and for the THSC model the discontinuities in both $c(\bar{r})$ and its 1st-order derivative at $\bar{r} = 1$ significantly affect its numerical accuracy.

To quantitatively compare the performance of our numerical approach with that of pyPRISM, we consider the HS model (*i.e.*, the THSC model with *N*=1), for which the PRISM-PY theory reduces to the OZ-PY theory and has the analytical result of

[*] E-mail: q.wang@colostate.edu

$c^*(\bar{r}) \equiv c(\bar{r}; m \to \infty, \bar{r}_c \to \infty) = -\left(2(1+2\eta)^2 - 3\eta(2+\eta)^2\bar{r} + \eta(1+2\eta)^2\bar{r}^3\right) / 2(1-\eta)^4$ for $0 \le \bar{r} < 1$

with the HS packing fraction $\eta \equiv \pi\bar{\rho}/6$, from which its 3D Fourier transform $\hat{c}^*(q)$ can be obtained analytically. We therefore take the numerical error as $\Delta\hat{c} \equiv \sqrt{\sum_{j=1}^{M}\left(\hat{c}(q_j;m) - \hat{c}^*(q_j)\right)^2 / M}$ with $\hat{c}(q_j;m)$ being the FFT of $c(\bar{r})$ obtained numerically; note that $\hat{c}_0$ (and $c(\bar{r}=0)$) is not given in pyPRISM. Fig. S1 shows that for pyPRISM $\Delta\hat{c}$ slowly decays towards 0 at a rate of $m^{-3/2}$, while for our approach it decays much faster with $m^{-9/2}$. Our Python script for calculating $\Delta\hat{c}$ using pyPRISM is given below:

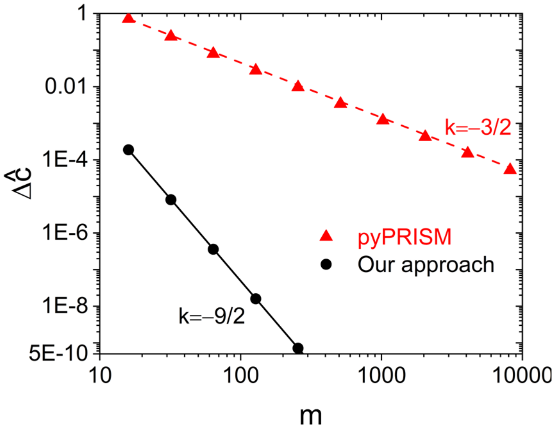

Figure S1. Logarithmic plot of the numerical errors $\Delta\hat{c}$ given by pyPRISM and our approach *vs.* the spatial discretization parameter m for the HS model at the dimensionless segment number density $\bar{\rho} = 0.6$, where the real-space cut-off $\bar{r}_c = 10$ and the k-value gives the slope of the corresponding straight line. See the main text for more details.

```
import pyPRISM
import numpy as np
import math

# Solve the OZ-PY equation for hard spheres
m=16
rc=10
rho=0.6
sys = pyPRISM.System(['A'],kT=1.0)
sys.domain = pyPRISM.Domain(dr=1/m,length=m*rc)
sys.diameter['A'] = 1.0
sys.potential['A','A'] = pyPRISM.potential.HardSphere()
sys.density['A'] = rho
sys.omega['A','A'] = pyPRISM.omega.SingleSite()
sys.closure['A','A'] = pyPRISM.closure.PercusYevick()
PRISM = sys.createPRISM()
PRISM.solve()
```

```
# Calculate c(q)
S = pyPRISM.calculate.structure_factor(PRISM)['A','A']
cq = (1-1/S)/rho

# Calculate rms deviation of c(q) from the analytical result
pi=math.pi
eta = pi*rho/6
dlt = 0
i = 0
for q in sys.domain.k:
    cqe = (-24*eta*(1+2*eta)**2 - 6*eta*(2+eta)**2*q*q \
          + (24*eta*(1+2*eta)**2 + 6*eta*(2-eta*(4+7*eta))*q*q \
             + (1-eta)**2*(2+eta)*q**4) * math.cos(q) \
          + 2*q*(12*eta*(1+2*eta)**2 - (1-6*eta+5*eta**3)*q*q) \
            * math.sin(q)) * 2*pi/((1-eta)**4*q**6)
    dlt += (cq[i]-cqe)**2
    i += 1
print (i, math.sqrt(dlt/i))
```

Table S1: List of Variables Used in the Main Text

$a = 15\varepsilon/\pi$	dimensionless excluded-volume interaction parameter between segments used in the dissipative particle dynamics potential
$\tilde{B}(q)$	normalized 3D Fourier transform of $\exp\left(-\beta u^{\mathrm{b}}(\overline{r})\right)$
$c(\overline{r})$	interchain direct segment pair correlation function
$\tilde{c}(\overline{r}) \equiv c(\overline{r}) - c_1 - c_1'(\overline{r}-1)$	an auxiliary function used for hard-sphere potential
$\hat{c}_0 \equiv \hat{c}(q_0)$	3D Fourier transform of $c(\overline{r})$ at q_0=0
$c_1 \equiv c(\overline{r} = 1^-)$	
$c_1' \equiv \left(\mathrm{d}c/\mathrm{d}\overline{r}\right)(\overline{r} = 1^-)$	
$C_0 \equiv N^2 \hat{c}_0 \sigma^3 / {R_{e,0}}^3$	a dimensionless parameter
$f(\overline{r})$	a radial function
$\hat{f}$	3D Fourier transform of $f(\overline{r})$
$h(\overline{r})$	interchain total segment pair correlation function
i, j	indices of segments in the system
k_B	Boltzmann constant
m	real-space discretization parameter
$M \equiv m\overline{r}_c$	
n	number of chains in the system
N	chain length
$\overline{\mathcal{N}} \equiv \left(n{R_{e,0}}^3/V\right)^2$	invariant degree of polymerization
P	pressure of the system
P_{ex}	excess (virial) pressure of the system due to interchain interaction
$\mathbf{q}$	dimensionless wavevector

$q=\|\mathbf{q}\|$	dimensionless wavenumber (in units of $1/\sigma$)
$\Delta q = m\pi/M$	
$q_c=m\pi$	reciprocal-space cut-off
$q_j\equiv j\Delta q$	$j=0,\ldots,M$
$\mathbf{r}$, $\mathbf{r}'$	spatial positions
$\bar{\mathbf{r}} \equiv \mathbf{r}/\sigma$	dimensionless spatial position
$\bar{r} \equiv r/\sigma$	dimensionless distance (in units of σ)
$\Delta\bar{r} \equiv 1/m$	
$\bar{r}_c$	dimensionless real-space cut-off (in units of σ)
$\bar{r}_i \equiv i\Delta\bar{r}$	$i=0,\ldots,M$
$R_{e,0} \equiv \sqrt{N-1}\sigma$	root-mean-square end-to-end distance of an ideal chain
T	thermodynamic temperature
$u_0(r)$	normalized non-bonded pair potential between segments
$u^{\mathrm{b}}(r)$	bonded potential between two segments
$u_{c,\mathrm{ex}}$	excess internal energy per chain of the system due to interchain interaction
$u^{\mathrm{DPD}}(r)$	non-bonded dissipative particle dynamics potential
$u^{\mathrm{HS}}(r)$	non-bonded hard-sphere potential
$u^{\mathrm{nb}}(r)$	non-bonded pair potential between segments
U^{nb}	total non-bonded interaction energy of the system
V	volume of the system
$\beta\equiv 1/k_BT$	
$\delta(r)$	Dirac δ-function
ε	dimensionless excluded-volume interaction parameter between segments
ε_c	maximum absolute value of the residual errors of the PY closure
$\phi(\mathbf{r})$	segment volume fraction at $\mathbf{r}$
$\gamma(\bar{r}) \equiv h(\bar{r}) - c(\bar{r})$	interchain indirect segment pair correlation function
$\tilde{\gamma}(q) \equiv \hat{\gamma} - \hat{\gamma}_c$	an auxiliary function used for the hard-sphere potential
$\hat{\gamma}_c \equiv \hat{\gamma}(q_c)$	
$\bar{\kappa}$	dimensionless excluded-volume interaction parameter used in the Edwards model
$\kappa_T \equiv -\left(\partial V/\partial P\right)_{n,\beta}/V$	isothermal compressibility
$\bar{\kappa}_T \equiv \rho_c\kappa_T/\beta$	normalized isothermal compressibility
$\rho\equiv nN/V$	number density of segments
$\bar{\rho} \equiv \rho\sigma^3$	dimensionless number density of segments
$\rho_c\equiv n/V$	number density of chains
σ	segment diameter (*i.e.*, the range of $u^{\mathrm{nb}}(r)$)
$\omega(\bar{r})$	normalized intrachain segment pair correlation function
$\hat{\omega}_{\mathrm{id}}$	3D Fourier transform of $\omega(\bar{r})$ for an ideal chain

References:

1. Martin, T. B.; Gartner, T. E.; Jones, R. L.; Snyder, C. R.; Jayaraman, A., pyPRISM: A Computational Tool for Liquid-State Theory Calculations of Macromolecular Materials. *Macromolecules* **2018,** *51* (8), 2906-2922.
